# Tromboelastography: variability and relation to conventional coagulation test in non-bleeding intensive care unit patients

**DOI:** 10.1186/s12871-015-0011-2

**Published:** 2015-03-08

**Authors:** Jørgen Holli Halset, Simon Wøhlert Hanssen, Aurora Espinosa, Pål Klepstad

**Affiliations:** 1 Faculty of Medicine, Norwegian University of Science and Technology, Trondheim, Norway; 2 Department of Immunology and Transfusion Medicine, St. Olav University Hospital, Trondheim, Norway; 3 Department of Anesthesiology and Intensive Care Medicine, St. Olav University Hospital, Trondheim, Norway; 4 Department of Circulation and Medical Imaging, Faculty of Medicine, Norwegian University of Science and Technology, Trondheim, Norway; 5 Department of Intensive Care Medicine, St. Olav University Hospital, P.O. box 3250 Sluppen, N-7006 Trondheim, Norway

**Keywords:** Critically ill, Coagulation, Tromboelastography

## Abstract

**Background:**

Intensive care unit (ICU) patients usually have abnormal biochemical and hematological laboratory test results as a consequence of organ dysfunction and underlying disease. Thromboelastography (TEG®) is a point-of-care laboratory analysis that gives an overview of several aspects of the coagulation process. In order to be able to perform a clinical interpretation of abnormal TEG® results the expected values from non-bleeding ICU patients should be known. The aim of this study is to report the normal variability observed in non-bleeding, non-transfused ICU patients.

**Methods:**

Adult ICU patients without bleeding in the last 24 hours, who had not received blood products within the last 24 hours, with no hematological diseases and no anticoagulation therapeutic treatment were included. Standard clinical chemistry tests, coagulation tests and TEG® were obtained. All results were reported in relation to standard reference values. TEG® values were compared with routine coagulation measurement using Spearman correlations.

**Results:**

We observed that the normal variability observed in non-bleeding, non-transfused ICU patients in this study included abnormally high TEG® values for maximum amplitude (MA) (73%). None of the patients showed MA results corresponding to hypocoagulability. Other coagulation tests were also changed with elevated D-Dimer, fibrinogen and APTT values, and a low ATIII value.

**Conclusion:**

In unselected ICU patients without bleeding or known factors that influence coagulation, a TEG® value of MA is often elevated suggesting hypercoagulability. This finding should be considered when interpreting TEG® observations obtained in ICU patients.

## Background

Intensive care unit (ICU) patients usually have abnormal biochemical and hematological test results as a consequence of organ dysfunction and underlying disease [1].

Coagulation abnormalities in the ICU setting can be multifactorial and must be identified early in order to start adequate treatment. In general, global coagulation tests such as international normalized ratio (INR), activated partial thromboplastin time (aPTT), platelet count and prothrombin time (PT) are used to monitor the coagulation system. These tests have several limitations as they are performed in plasma or serum, and do not reflect the *in vivo* haemostatic process or the function of the cell components in the coagulation system. New point-of-care methods, such as rotational thomboelastometry (ROTEM®) and thromboleastography (TEG®), have increasingly been used in the last decade to assess the haemostatic function. These tests have many advantages compared to routine coagulation tests as they give an overview of several aspects of the coagulation process, from the initial thrombin formation to the clot lysis.

TEG® was developed by Hartert in 1948 and it measures the viscoelastic changes that occur during coagulation *in vitro*. The instrument generates a graphical representation that reflects the initial fibrin-platelet interaction, platelet aggregation, clot strength, rate of fibrin polymerization, and fibrinolysis [2]. TEG® is a well-established tool for monitoring coagulation during and after liver transplantation and cardiac surgery [3]. Several studies show that the use of TEG®-based algorithms reduces transfusion requirements in cardiac surgery [3,4].

The reference values for the TEG® are based on results from healthy individuals and they might not be directly applicable to all patients [5-7]. Previous studies have investigated the TEG® results in ICU patients with sepsis, but to our knowledge, there are no studies where TEG® analysis has been performed in a general ICU patient population without active bleeding or a recent blood transfusion history [6,8].

In order to be able to perform a clinical interpretation of abnormal TEG® results in the ICU one of the requirements is to establish the expected values from non-bleeding ICU patients. Therefore, the aim of this study is to report the normal variability observed in non-bleeding, non-transfused ICU patients and to compare TEG® findings with other coagulation tests.

## Methods

### Patients and setting

This study was performed at the 9-bed mixed case ICU at St. Olav University Hospital, Trondheim, Norway, in the period June 2012 to September 2013. In this period it was decided to obtain conventional coagulation tests and point of care coagulation test (TEG®) as part of routine care. Routinely an extended set of analyses including coagulation tests is obtained from all patients in the unit twice weekly (Monday and Thursday). As a part of a quality assurance initiative test results in this period were collected and analyzed retrospectively. Inclusion criteria for the present analyses were ICU patients older than 18 years with a length of stay more than 12 hours. Patients were excluded by the following criteria: known bleeding exceeding 300 ml the last 24 hours before sampling, hemoglobin (Hgb) -variability of ≥ 2 g/dl within 6 hours prior to or 6 hours after the sample was taken, any transfusion of blood products (red blood cells. fresh frozen plasma. platelet concentrate or others) the last 24 hours before sampling, known bleeding disorders (e.g. hemophilia or other factor deficiencies) or other diseases expected to affect coagulation (e.g. hematological malignancies), and use of anticoagulants (except enoxaparin ≤40 mg/day and/or acetylsalicylic acid ≤300 mg/day).

### Clinical variables

All data were collected from the patients’ hospital records. Variables obtained included demographic data, primary diagnosis during the ICU-stay, Simplified Acute Physiology Score II (SAPS II) [9], Sequential Organ Failure Assessment (SOFA) score [10] (for the day of the TEG®-sample) and all medications.

### Blood analyses

Arterial blood samples for the kaolin TEG®-analysis were drawn from an arterial line into a tube containing 3.2% sodium-citrate and were analyzed within 2 hours according to the manufacturers recommendations [11] after recalcification with 20 μl 0.2 M CaCl_2_. A total of five TEG®**-**variables were recorded; reaction time (R), k-time (K), maximum amplitude (MA), angle (α) and the calculated G-value. Briefly, R represents the time until initial fibrin formation, K and α represent speed and kinetics of clot formation, MA represents the maximal clot strength and stability of the fibrin clot and G is calculated from the MA providing a more sensitive parameter for changes in clot strength [11].

Hemoglobin, white blood cell count, creatinine, bilirubin, sodium, potassium, ionized calcium, magnesium, phosphate, albumin, C-reactive protein (CRP), platelet count, INR, d-dimer, fibrinogen, antithrombin-III and aPTT were obtained using the hospitals standard analytical methods. Due to practical concerns the blood samples for the TEG® were obtained two hours later than the other tests.

### Analysis/statistics

IBM SPSS version 21 was used for all statistical analysis. For biochemistry and TEG®-values. the median and interquartile range were used for descriptive data. All TEG®-values were also compared to the manufacturers reference values [11], and categorized as below, within or above these reference values.

Spearman correlations were performed for all TEG®-variables according to age and gender. Correlation analysis was performed between time from sampling and TEG®-parameters. We also analyzed possible correlations between TEG®-parameters with other coagulation-parameters, and clinical chemistry tests expected to affect coagulation. This is a descriptive study and no adjustments for multiple testing were performed. Given the multiple statistical tests, we considered only p-values < 0.01 significant in order to diminish risk of type-I errors.

### Ethics

The Regional Committee for Medical and Health Research Ethics Norwegian Central approved the study and because samples were obtained for clinical routine use waived the need for an informed consent.

## Results

We included 82 patients (61% male, median age 61 years) in the study. The most frequent diagnoses for ICU admission were respiratory failure, infections, trauma and non-traumatic CNS diseases (Table 1). The median SAPS II score was 38 (IQR 29–49) and median total SOFA score was 6 (IQR 4–8). An overview of the patients’ medications and results from the routine biochemistry tests is shown in Table 1.

**Table 1:** **Demographic data and clinical chemistry results**

		N*	Median	Interquartile range
**Age**			61	47-69
**Gender (male)**		50 (61%)		
**Diagnostic category**	Trauma	11 (13.4%)		
	Circulation	7 (8.5%)		
	Respiration	19 (23.2%)		
	Post-operative	9 (11.0%)		
	Infection/sepsis	12 (14.6%)		
	Gastrointestinal	5 (6.1%)		
	CNS non-trauma	11 (13.4%)		
	Others	8 (9.8%)		
**SAPS II**			38	29-49
**Total SOFA score**			6	4-8
**Number of organ failures**	0	18		
	1	29		
	2	24		
	3	10		
	≥4	0		
**Medications**	Beta blockers	16 (19.5%)		
	Calcium blockers	6 (7.3%)		
	Nitro-preperations	1 (1.2%)		
	Diuretics	46 (56.1%)		
	Enoxaprin low dose	60 (73.2%)		
	ASA low dose	4 (4.9%)		
	Vasoactive substances	60 (73.2%)		
	Insulin	49 (59.8%)		
	Propofol	41 (50.0%)		
	Benzodiazepines	49 (59.8%)		
	Opioids	71 (86.6%)		
	Antibiotics	67 (81.7%)		
Clinical chemistry**	Hemoglobin (g/dL)		9.9	9.2-11.1
	White blood cell count (10^9^/L)		12.8	9.7-16.5
	Creatinine (μmol/L)		63	44-108
	Bilirubin (μmol/L)		6	4-12
	Sodium (mmol/L)		138	135-141
	Potassium (mmol/L)		3.98	3.72-4.23
	Calcium ionized (mmol/L)		1.14	1.10-1.18
	Magnesium (mmol/L)		0.81	0.73-0.88
	Phosphate (mmol/L)		0.96	0.75-1.08
	Albumin (g/L)		24	20-27
	CRP (mg/L)		121	67-201

*N = number of patients (percent of study population). CNS = central nervous system. SAPS II = Simplified Acute Physiology Score II. SOFA = Sequential Organ Failure Assessment score. ASA = acetylsalicylic acid. WBC = white blood count. CRP = C reactive protein. **Clinical chemistry reference values: Hb: 11.7 - 15.3 (female) and 13.4 - 17.0 (male). WBC: 3.7 – 10 . Creatinine: 45–90 (female) and 60 – 105 (male). Bilirubin (total): 5 – 25. Sodium: 137 – 145. Potassium: 3.6 – 4.6. Calcium ionized: 1.18 – 1.32. Magnesium: 0.71 – 0.94. Phosphate: 0.85 – 1.85 (female) and 0.75 - 1.35 (male). Albumin: 34 – 45. CRP: <5.

Median platelet count and INR were within normal values. Median fibrinogen and D-dimer were higher than reference values and median ATIII values were low compared with normal values. The numbers of observations below and above reference values are given in Table 2. MA and G values were in 73% of the cases above the manufacturer’s reference range (Table 2). For the rest of the TEG®-parameters the results were within reference values in most patients (Table 2).

**Table 2:** **Results of TEG® and other coagulation tests**

	Number	Results	Number of tests in relation to normal values*.**		
			Above	Within	Below
**TEG®**					
R (min)	82	5.75 (4.78-6.83)	12	70	0
K (min)	82	1.45 (1.20-1.90)	5	71	6
α (deg)	82	69.8 (63.9-73.3)	4	71	7
MA (mm)	82	73.4 (68.3-78.2)	60	22	0
G (dyn/cm^2^)	82	13.8 (10.8-18.0)	60	22	0
**Other coagulation results**					
Platelets (10^9^/L)	82	216 (163–336)	14	52	16
INR	80	1.2 (1.1-1.3)	25	55	0
D-dimer (mg/L)	69	4.0 (2.5-8.6)	67	2	
Fibrinogen (g/L)	71	6.5 (4.7-7.6)	61	10	0
AT III (%)	65	82.0 (68.0-100.5)	3	23	39
APTT (sec)	70	38.0 (34.0-43.0)	13	56	1

All results given In median (interquartile range) *Manufacturers reference values. TEG® 5000: R: 2–8. K: 1–3. α: 55–78. MA: 51–69. G: 4.6-10.9.

**Clinical chemistry reference values: platelets: 145–390. International normalised ratio (INR): 0.9-1.2. D-dimer < 0.5. Fibrinogen: 2.0-4.0. antithrombin III (AT-III): 89–118. activated partial thromboplastin time (APTT): 30–44.

There was no significant correlation between age or gender with TEG® values. The median time from sampling to analyses was 50 minutes (IQR: 33–82). There were no significant correlations between time from sampling to analysis and TEG® values.

Correlations for TEG®-variables with standard coagulation tests and hemoglobin are shown in Table 3. We observed strong correlations between MA and the platelet count (r = 0.680, p > 0.001) and fibrinogen (r = 0.619, p < 0.001). Hgb correlated negatively with MA (r = −0.379, p < 0.001). G shoved the same pattern of correlations as MA. K was correlated with APTT (r = 0.368, p = 0.003) and Hgb (r = 0.368, p = 0.001), and negatively correlated with platelet number (r = −0-450, p < 0.001). α correlated with platelet number (r = 0.475, p < 0.001) and had negative correlations with APTT (r = −0354, p = 0.003) and Hgb (r = −0.414, p < 0.001). R was only correlated with APTT (r = 0.339, p < 0.001).

**Table 3:** **Correlations between TEG®-values. coagulation tests and Hb**

		R	K	α	MA	G
Platelets	Correlation	−0.180	**−0.450**	**0.475**	**0.680**	**0.882**
	Sig. (2-tailed)	0.106	**<0.001**	**<0.001**	**<0.001**	**<0.001**
INR	Correlation	0.0.80	0.207	−0.200	−0.168	−0.166
	Sig. (2-tailed)	0.478	0066	0.076	0.136	0.140
D-dimer	Correlation	0.0.22	−0.033	0.068	0.246	0.244
	Sig. (2-tailed)	0.858	0785	0.579	0.042	0.0.44
Fibrinogen	Correlation	0.004	−0.160	0205	**0.619**	**0.617**
	Sig. (2-tailed)	0.977	0.183	0.086	**<0.001**	**<0.001**
AT-III	Correlation	0.027	−0.241	0.246	0.279	0.280
	Sig. (2-tailed)	0.832	0.054	0.0.48	0.024	0.024
APTT	Correlation	**0.339**	**0.348**	**−0.354**	−0.075	−0.076
	Sig. (2-tailed)	**0.001**	**0.003**	**0.003**	0.538	0.531
Hemoglobin	Correlation	0.125	**0.368**	**−0.414**	**−0.379**	**−0.377**
	Sig. (2-tailed)	0.267	**0.001**	**0 < .001**	**<0.001**	**0.001**

All correlations are Spearmans correlations. Bold text represents correlations with a p-value < 0.01.

## Discussion

We observed that the normal variability in non-bleeding, non-transfused ICU patients included in this study included TEG® values for MA and G higher than reference values. Other coagulation tests were also abnormal with D-Dimer, fibrinogen, APTT values higher than reference values and ATIII lower than reference values.

We observed that approximately 73% of the patients had a TEG® profile suggesting hypercoagulability reflected by a high MA value [6,12]. None of the patients had a MA value corresponding to hypocoagulability. This finding differs from a study by Ostrowski et al. who observed that 30% of septic ICU patients were hypocoagulable, 22% were hypercoagulable and 48% were normocoagulable [6]. The authors suggested that these findings reflected the severity of sepsis and that TEG® values were independently associated with 28-day mortality [6]. Massion et al. also reported the presence of a hypocoagulable state caused by hyperfibrinolysis measured with thromboelastometry and standard coagulation tests in patients diagnosed with septic shock [13]. The differences between these studies and our results are not exclusively explained by different case loads, septic patients versus a mixed ICU population, because many patients also in our cohort were septic.

We did not observe any of the correlations of TEG® values with age, gender or time from sampling to analysis that are described in other studies in other patient populations [14-16]. This lack of associations may suggest that the many clinical factors influencing coagulation in ICU patients minimize the relative contribution to variability from some of the factors relevant in a healthy population [14-16]. In our population the median hemoglobin serum concentration was lower than reference values and hemoglobin serum concentration correlated negatively with MA. This is in accordance with other studies, where low hemoglobin values are associated with high MA and R values *in vitro* [17,18]. Ostrowski et al. showed that fibrinogen but not platelets contributed to TEG® clot strength in hypocoagulable and hypercoagulable patients, whereas both platelets and fibrinogen contributed independently to TEG® clot strength in normocoagulable patients [6]. In our study we observed that both fibrinogen and platelet count were associated with MA.

Critically ill patients have many similar physiological disturbances, often elicited by a non-specific systemic inflammatory response syndrome, regardless of the underlying disease. In this study, we selected an ICU population without on-going bleeding and with no specific factors related to a haematological disease or therapeutic anticoagulation therapy. Furthermore, different from previous studies, we did not restrict the analyses to patients with sepsis. Thus, the TEG® values in our study population might be representative for ICU patients in general. ICU patient case-mix may be different in other ICU cohorts, in the ICU cohort in this study the severity of disease was high as illustrated by the median SAPS II score of 38. We observed that the majority of the study population had MA and G values higher than reference values, which would suggest hypercoagulability. Other TEG® parameters were mostly within references values, while traditional coagulation measures showed, as expected, high values of D-dimer and fibrinogen and low values of AT-III. This finding has implications for the interpretation of TEG® findings in bleeding ICU patients in order to differentiate between findings expected to be associated with the cause of bleeding and findings that simply can be a part of the inflammatory process. Thus, TEG® findings may be interpreted different in ICU patient than in patients with acute bleeding due to the expected changes in the ICU patients with a multi-organ failure.

We recognize some limitations in this study. First, we have no data regarding the patients that were not included. However, we do not know of any systematic bias in the inclusions. Second, the results may be different in ICU populations with a different case-mix. Thirdly, only one sample was obtained from each patient, thus inter-test-variability could not be analysed. Finally, as this is a study performed in the ICU all samples are collected from an arterial line, which may be different from analyses performed in venous blood.

## Conclusion

ICU patients without bleeding or known factors that influence coagulation have often high TEG® values of MA and G suggesting hypercoagulability. This finding should be considered when interpreting TEG® observations obtained in ICU patients.

### Key messages

- Hypercoagulability showed by high MA and G values at tromboelastography (TEG®) is frequent in an unselected ICU population with no ongoing bleeding.
- Other tromboelastography (TEG®) values than MA and G are usually within normal values in an unselected ICU population with no ongoing bleeding.

## Abbreviations

Α: Angle
APTT: Activated partial thromboplastin time
ATIII: Anti-thrombin III
CaCl_2_: Calciumcloride
CRP: C-reactive protein
Hgb: Hemoglobin
ICU: Intensive care unit
INR: International normalized ratio
K: k-time
MA: Maximum amplitude
PT: Prothrombin time
R: Reaction time
ROTEM: Rotational thomboelastometry
SAPS II: Simplified acute physiology score II
SOFA: Sequential organ failure assessment
TEG®: Thromboelastography
